# Azithromycin is able to control *Toxoplasma gondii* infection in human villous explants

**DOI:** 10.1186/1479-5876-12-132

**Published:** 2014-05-19

**Authors:** Letícia S Castro-Filice, Bellisa F Barbosa, Mariana B Angeloni, Neide M Silva, Angelica O Gomes, Celene M O S Alves, Deise A O Silva, Olindo A Martins-Filho, Maria C Santos, José R Mineo, Eloisa A V Ferro

**Affiliations:** 1 Laboratory of Immunophysiology of Reproduction, Institute of Biomedical Sciences, Universidade Federal de Uberlândia, Av. Pará 1720, Uberlândia, MG 38400-902, Brazil; 2 Laboratory of Immunopathology, Institute of Biomedical Sciences, Universidade Federal de Uberlândia, Av. Pará 1720, Uberlândia, MG 38400-902, Brazil; 3 Laboratory of Chagas Disease, René Rachou Research Center, Fundação Oswaldo Cruz, Belo Horizonte, MG 30190-002, Brazil; 4 Department of Gynecology and Obstetrics, Faculty of Medicine, Universidade Federal de Uberlândia, Uberlândia, MG, Brazil

**Keywords:** Azithromycin, Human placental villi, Trophoblast, Cytokines, Sex hormones, *Toxoplasma gondii*

## Abstract

**Background:**

Although *Toxoplasma gondii* infection is normally asymptomatic, severe cases of toxoplasmosis may occur in immunosuppressed patients or congenitally infected newborns. When a fetal infection is established, the recommended treatment is a combination of pyrimethamine, sulfadiazine and folinic acid (PSA). The aim of the present study was to evaluate the efficacy of azithromycin to control *T. gondii* infection in human villous explants.

**Methods:**

Cultures of third trimester human villous explants were infected with *T. gondii* and simultaneously treated with either PSA or azithromycin. Proliferation of *T. gondii*, as well as production of cytokines and hormones by chorionic villous explants, was analyzed.

**Results:**

Treatment with either azithromycin or PSA was able to control *T. gondii* infection in villous explants. After azithromycin or PSA treatment, TNF-α, IL-17A or TGF-β1 levels secreted by infected villous explants did not present significant differences. However, PSA-treated villous explants had decreased levels of IL-10 and increased IL-12 levels, while treatment with azithromycin increased production of IL-6. Additionally, *T. gondii*-infected villous explants increased secretion of estradiol, progesterone and HCG + β, while treatments with azithromycin or PSA reduced secretion of these hormones concurrently with decrease of parasite load.

**Conclusions:**

In conclusion, these results suggest that azithromycin may be defined as an effective alternative drug to control *T. gondii* infection at the fetal-maternal interface.

## Introduction

*Toxoplasma gondii* is an obligate intracellular protozoan parasite that infects a wide range of hosts, including humans. During pregnancy, primary infection can result in the vertical transmission of *T. gondii* tachyzoites, potentially having severe consequences for the fetus, such as retinochoroiditis as well as hydrocephalus and intracranial calcification [1]. The risk of fetal infection by *T. gondii* increases as pregnancy progresses [2,3]. Nevertheless, the consequences of fetal infection are more severe the earlier infection occurs during pregnancy [4].

A combination of pyrimethamine, sulfadiazine and folinic acid (PSA) or spiramycin are standard care for treatment of toxoplasmosis in cases of fetal infection [5,6]. Treatment with spiramycin is administered immediately after diagnosis of maternal infection [7], and since this macrolide does not cross placenta, it is not suitable for treatment of fetal infections [8]. PSA is the recommended combination for pregnant women treatment who acquire the infection after 18 weeks of gestation. However, pyrimethamine is potentially teratogenic and should not be used in the first trimester of pregnancy [8]. Pyrimethamine is a folic acid antagonist and its use in pregnancy has been associated with increased risk of neural tube defects [9]. This drug is potentially toxic, usually causing gradual dose-related bone marrow depression, chromosomal damage and mutagenicity [9,10]. Folinic acid is used for reduction and prevention of hematological toxicities caused by pyrimethamine [8]. Alternative drugs for treatment of toxoplasmosis, such as trimethoprim-sulfamethoxazole or clindamycin, have shown activity *in vitro* and in mouse models, but clinical studies to establish their efficacy have not been conclusive [10]. Thus, there is an urgent need for more effective and non-toxic chemotherapeutic agents and novel drug targets to be identified for treatment of *T. gondii* infection during pregnancy.

Azithromycin is a semisynthetic azalide antibiotic that is structurally related to erythromycin, but has a broader spectrum of antibacterial activity and a more favorable pharmacokinetic profile. Furthermore, administration is required only once per day [11-13]. Azithromycin also inhibits protein synthesis in the plasmodial apicoplast and, thus, has activity against both *Plasmodium falciparum* and *P. vivax*[12]*.* Azithromycin is widely used for the treatment of community-acquired pneumonia and chlamydia during pregnancy, and has been safely administered in all trimesters of gestation [14,15]. Studies have shown that animals receiving 60 times the recommended dose of azithromycin for humans do not have decreased fertility or demonstrated harmful effects on the fetus [16]. Given that azithromycin is considered safe during pregnancy and the fact that it may have activity against pathogens, such as *Plasmodium* and *Toxoplasma*, it has been suggested as a candidate for intermittent preventive treatment during pregnancy [7,17]. Previous studies have shown that azithromycin treatment in pregnant *Calomys callosus* and human trophoblast cells (lineage BeWo) was able to control *T. gondii* infection, suggesting that it may be an alternative drug for prevention of congenital infection [18,19]. Additionally, azithromycin has anti-inflammatory properties through modulating the production of proinflammatory cytokines that are produced during *T. gondii* infection [20].

Thus, the aim of the present study was to evaluate the efficacy of azithromycin in the control of *T. gondii* infection in third trimester human villous explants cultures compared to traditional therapy (PSA).

## Methods

### Placental samples

Placental tissues were obtained from 24 women after elective cesarean section deliveries (36 to 40 weeks of pregnancy). Exclusion criteria included pre-eclampsia, chronic hypertension, infectious disease including toxoplasmosis, chorioamnionitis, chronic renal disease, cardiac disease, connective tissue disease, pre-existing diabetes mellitus and gestational diabetes mellitus. Placental tissues were placed in ice-cold sterile phosphate-buffered saline (PBS), pH 7.2, to remove excess blood then aseptically dissected using a stereomicroscope to remove endometrial tissue and fetal membranes up to 1 h after collection. Floating terminal chorionic villous explants containing five to seven free tips per explant were collected as described previously [21,22]. Explants were added to 96-well plates (one per well) and cultured in complete medium containing RPMI 1640 medium (Cultilab, Campinas, SP, Brazil) supplemented with 10% fetal bovine serum (FBS) (Cultilab), 100 U/mL penicillin, and 100 μg/mL streptomycin (Sigma-Aldrich Co., St. Louis, MO, USA) for 24 h at 37°C and 5% CO_2_.

### Parasites

Tachyzoites from *T. gondii* 2 F1 strain, which constitutively express cytoplasmic β-galactosidase and are derived from the RH strain, were maintained by serial passages in BeWo cells in RPMI medium supplemented with penicillin, streptomycin and 2% FBS at 37°C and 5% CO_2_. *T. gondii* 2 F1 was a gift from Dr. Vern Carruthers, Medicine School of Michigan University (USA).

### Antibiotics

Macrolide azithromycin (Biofarma, Uberlândia, MG, Brazil) or a drug combination consisting of pyrimethamine (Daraprim® - Farmoquímica, Rio de Janeiro, RJ, Brazil), sulfadiazine (Suladrin®- Laboratório Catarinense, Joinville, SC, Brazil) and folinic acid (Genix, Anápolis, GO, Brazil) (PSA) were dissolved in culture medium to a concentration of 5000 μg/mL (stock solution). Different drug concentrations were prepared by diluting the stock solution and used for treatment of villous explants.

### Treatments

First, villous explants were infected with *T. gondii* 2 F1 strain tachyzoites (1 × 10^6^ parasites per well) and incubated for 48 h. Next, villous explants were washed with complete medium to remove non-adhered parasites and treated for an additional 24 h with different concentrations of azithromycin (200, 1000 or 5000 μg/mL) or PSA as follows: 200, 1000 or 5000 μg/mL for pyrimethamine; 150, 750 or 3750 μg/mL for sulfadiazine; and 30, 150 or 750 μg/mL for folinic acid, as previously established [23]. Uninfected/untreated villous explants or infected/untreated villous explants (NT) were cultured with complete medium only and included as experimental controls. After, culture supernatants were collected and stored at -70°C for measurement of cytokines or nitrite at a later time. Villous explants were collected for morphological analysis, immunohistochemistry, or analysis for *T. gondii* intracellular proliferation using a colorimetric β-galactosidase assay as previously described [24]. *T. gondii* intracellular proliferation data were expressed as the number of tachyzoites calculated in relation to the standard curve of 2 F1 strain tachyzoites ranging from 1 × 10^6^ to 15.625 × 10^3^ total parasites.

In another set of experiments, parasites were pre-treated with antibiotics. Briefly, 2 F1 strain tachyzoites were treated with different concentrations of azithromycin (50, 100, 200, 1000 μg/mL) or PSA (50, 100, 200 or 1000 μg/mL for pyrimethamine; 37.5, 75, 150 or 750 μg/mL for sulfadiazine and 7.5, 15, 30 or 150 μg/mL for folinic acid), diluted in culture complete medium for 1 h at 37°C in a CO_2_ incubator. Afterwards, parasites were placed in 96-well tissue culture plates containing previously collected villous explants. Untreated parasites (NTp) remained for 1 h in complete medium under the same conditions as treated parasites and were used to infect villous explants. After 72 h, villous explants were collected for *T. gondii* proliferation assays according Teo et al. [24].

### *T. gondii* intracellular proliferation assay

The villous explants were submitted to a colorimetric β-galactosidase assay in order to analyze the *T. gondii* intracellular proliferation [24] with minor modifications. Briefly, villous explants were homogenized in radioimmunoprecipitation assay buffer (RIPA) [50 mmol/L Tris hydrochloride, 150 mmol/L NaCl, 1% (v/v) Triton X-100, 1% (w/v) sodium deoxycholate, and 0.1% (w/v) SDS; pH 7.5] plus protease inhibitor cocktail tablets (Roche Diagnostics GmbH, Mannheim, Germany). The homogenate was centrifuged at 15,000 x *g* for 15 minutes at 4°C, the supernatants were used for protein content measurement using the Bradford method [25] and 100 μg/mL of total protein was used to perform the colorimetric β-galactosidase assay. Then, the total protein (100 μg/mL) was incubated with 160 μL of assay buffer (100 mM phosphate buffer, pH 7.3, 102 mM mercaptoethanol and 9 mM MgCl2) and 40 μL 6.25 mM CPRG (chlorophenol red-β-D-galactopyranoside; Roche, Indianapolis, IN) for 30 min. The enzymatic activity of β-galactosidase was measured at an absorbance of 570 nm using a kinetic plate reader (Titertek Multiskan Plus, Flow Laboratories, McLean, VA, USA).

### Toxicity assay

Culture supernatants were collected and measurement of lactate dehydrogenase (LDH) was immediately performed in order to determine cytotoxicity of tested drugs to infected human villous explants. Measurement of LDH released from the cytosol of lysed cell membranes into the supernatant was performed using a commercial diagnostic kit according to manufacturer instructions (LDH Liquiform, LabtestDiagnostica S.A., Lagoa Santa, MG, Brazil). The method used is based on consumption and decrease of absorption of NADH at 340 nm, which was measured in a DU-70 spectrophotometer (Beckman, Brea, CA, USA) for 3 min at 37°C. The data were expressed in U/mL of enzyme activity. The negative control (uninfected control) was determined by spontaneous LDH released from uninfected villous explants. Maximum LDH released, defined as positive control (total cell lysis), was determined by adding 0.2% Triton X-100 to explants. In parallel, cytokine and hormone levels were measured in supernatants. The doses of 200 and 1000 μg/mL for azithromycin or pyrimethamine; 150 and 750 μg/mL for sulfadiazine; and 30 to 150 μg/mL for folinic acid were chosen for this latter purpose according to toxicity results. All subsequent experiments were performed using these drug concentrations. Hereafter, PSA concentrations will be denoted based on the concentration of pyrimethamine.

### Morphological analysis and immunohistochemistry

To verify the integrity of villous explants and immunolocalization of parasites, fragments of placental explants were fixed in 10% buffered formalin, dehydrated in increasing alcohol concentrations, and embedded in paraffin. Sections with 4 μm were placed on glass slides and subjected to immunohistochemical analysis [22]. For antigen retrieval, sections were covered with trypsin solution (0.05% trypsin and 0.1% calcium chloride (Sigma-Aldrich Co.) for 30 min at 37°C. Explant sections were incubated in 5% acetic acid at room temperature in order to block endogenous phosphatase activity. Next, sections were incubated overnight at 4°C with hyperimmune rabbit anti-*T. gondii* serum (1:100). Biotinylated goat anti-rabbit IgG (1:600, Sigma-Aldrich Co.) was used as secondary antibody, and the reaction was developed with fast red naphthol (Sigma-Aldrich Co.). Samples were counterstained with Harris’s hematoxylin and examined under a light microscope (BX40, Olympus, Tokyo, Japan).

### Cytokine and nitrite assays

Villous explant culture supernatants were analyzed for IL-12, TNF-α, IL-10 and TGF-β1 concentrations using a sandwich ELISA according to the manufacturer’s instructions (R&D Systems, BD Biosciences, San Diego, CA, USA). Cytokine concentration was determined by extrapolation from a standard curve obtained from known concentrations of the respective recombinant cytokines. Optical density was measured at 450 nm with a microplate reader (Titertek Multiskan Plus, Flow Laboratories, McLean, VA, USA). The limit of detection of the assays was 7.8 pg/mL for both TNF- α and IL-12, 62.5 pg/mL for IL-10, and 31.3 pg/mL for TGF- β1.

Additionally, human cytokines (IL-2, IFN-γ, IL-6, IL-4 and IL-17A) were quantified using a cytometric bead array^TM^ (CBA; BD Biosciences) according to the manufacturer’s instructions. Samples were analyzed using a BD^TM^ flow cytometer (FACSCalibur, BD Biosciences), and data were calculated using specialized BD^TM^ Cell Quest and CBA software (BD Biosciences).

Also, supernatants were analyzed by Griess method [25] in order to measure nitrite. Briefly, the supernatants were added in triplicate to 96-well plates and mixed 1:1 with 1% sulfanilamide dihydrochloride and 0.1% naphthylenediamide dihydrochloride in 2.5% H3PO4. Absorbance was read in a plate reader at 570 nm, and concentration was determined with reference to a standard curve of sodium nitrite with concentrations ranging from 5 to 200 μmol/L.

### Hormone measurement

Villous explant culture supernatants were also analyzed for concentrations of estradiol, progesterone and HCG + β by an electrochemiluminescence immunoassay (ECLIA) intended for use on an automated analyzer Cobas e 411 (Roche Diagnostics GmbH, Mannhein, Germany), according to the manufacturer’s instructions. The limit of detection of the assays was 5.0 pg/mL for estradiol, 0.03 ng/mL for progesterone and 0.01mUI/mL for HCG + β.

### Measurement of villous explant protein content

Frozen villous explants were homogenized in radioimmunoprecipitation assay buffer (50 mM Tris hydrochloride, 150 mM NaCl, 1% (v/v) Triton X-100, 1% (w/v) sodium deoxycholate and 0.1% (w/v) SDS; pH 7.5) plus a protease inhibitor cocktail (Roche Diagnostics). Homogenate was centrifuged at 15,000 × *g* for 15 min at 4°C. Supernatants were used for protein content measurement using the Bradford method [26]. To normalize explants from different sizes, a ratio between cytokine production (pg/mL) and its corresponding protein content (mg/mL) was calculated, and the data were expressed in picograms/milligram (pg/mg) as previously proposed [21]. Likewise, concentrations of estradiol, progesterone, and HCG + β were normalized according to the corresponding protein content of villous explants, and the data were expressed in pg/mg, ng/mg and mIU/mg, respectively.

### Statistical analysis

Data were expressed as mean ± standard error of mean (SEM), and the differences between groups were determined using ANOVA (with correction for multiple comparisons via the Bonferroni method) or a Student *t*-test, when appropriate (GraphPad Prism Sofware 5.0 GraphPad Software, Inc., San Diego, CA, USA). Differences were considered statistically significant when P < 0.05. Six independent experiments were performed in triplicate using 24 placental tissues.

## Results

### Antibiotic cytotoxicity is dose dependent

**Figure 1:**
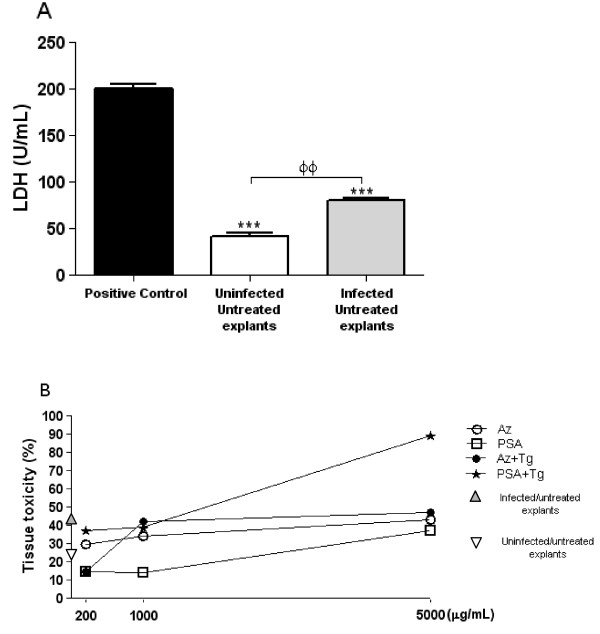
**Analysis of cytotoxicity in human villous explants.** Villous explant supernatants were collected and lactate dehydrogenase (LDH) activity was measured using the LDH Liquiform kit. The positive control was established by adding 0.2% Triton X-100 **(A)**. Human villous explants were infected or not by *T. gondii* and treated with increasing concentrations of azithromycin or PSA for 24 h and the percentage of tissue toxicity was analyzed **(B)**. Data are expressed as mean ± SEM. Significant differences were analyzed by ANOVA and Bonferroni multiple comparison post hoc test. ^***^P *<* 0.001 in relation to the positive control; ^ΦΦ^ P *<*0.01 between uninfected and infected villous explants.

The degree of cytotoxicity of different doses of antibiotics was evaluated by measuring LDH release in villous explant culture supernatants. *T. gondii* infection induced greater LDH release and increased cytotoxicity compared to uninfected villous explants (Figure 1A; ANOVA, P < 0.001). Both drugs at concentrations of 200 or 1000 μg/mL did not cause significant cytotoxicity in villous explants compared to untreated villous explants, regardless of *T. gondii* infection (Figure 1B). However, infected villous explants showed a large increase in cytotoxicity when treated with 5000 μg/mL of PSA compared to untreated villous explants (Figure 1B). Therefore, the concentrations of 200 and 1000 μg/mL were used in further experiments.

### Azithromycin and PSA are able to control *T. gondii* infection in villous explants

**Figure 2:**
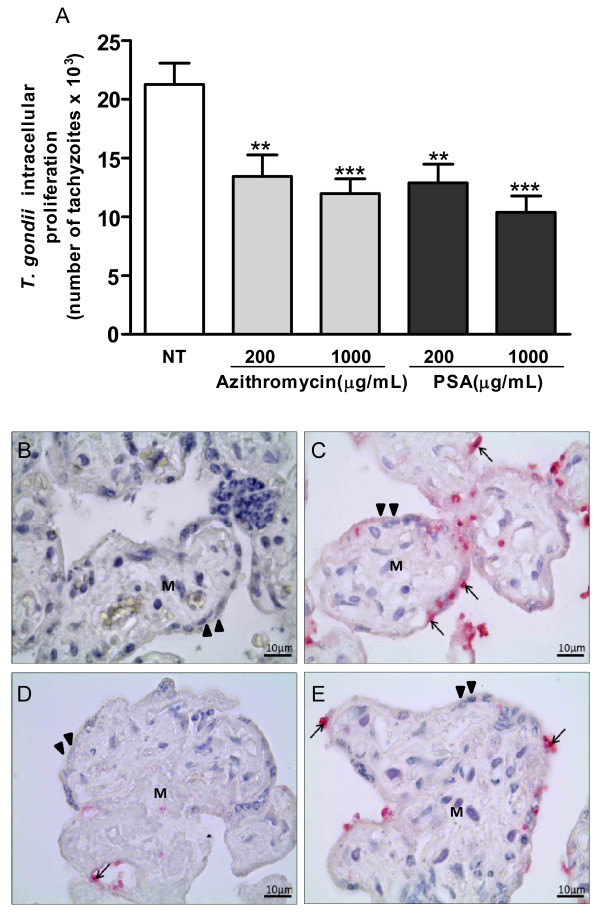
***T. gondii *intracellular proliferation in villous explants treated with azithromycin or PSA.** Villous explants were infected and treated for 72 h with azithromycin or PSA at different concentrations. *T. gondii* proliferation was measured by a colorimetric β-galactosidase assay **(A)**. The bars represent mean ± SEM. Data were analyzed by ANOVA and Bonferroni multiple comparison post hoc test. Significant differences in comparison with untreated villous explants are labeled (^***^P *<* 0.001; ^**^P *<*0.01). Representative photomicrographs of human villous explants infected or not with *T. gondii*: uninfected human villous explants as a negative control **(B)**, arrows indicate tachyzoites in untreated human villous explants **(C)**, azithromycin-treated **(D)** and PSA-treated **(E)** human villous explants. Arrowheads indicate syncytiotrophoblast layers, and **M** indicates mesenchymal tissue. Counterstaining with Mayer’s hematoxylin was performed. Scale bar: 10 μm.

Treatment with either azithromycin or PSA significantly reduced intracellular proliferation of *T. gondii* at both tested concentrations (200 and 1000 μg/mL) compared to untreated villous explants (Figure 2A; ANOVA, P < 0.001). In addition, treatment with either drug did not induce morphological alterations in villous explants (Figure 2, B to E). It was observed syncytiotrophoblast covering the chorionic villus, the cytotrophoblast and mesenchymal tissue.

### Treatment with azithromycin increases IL-6 production by *T. gondii*-infected villous explants

**Figure 3:**
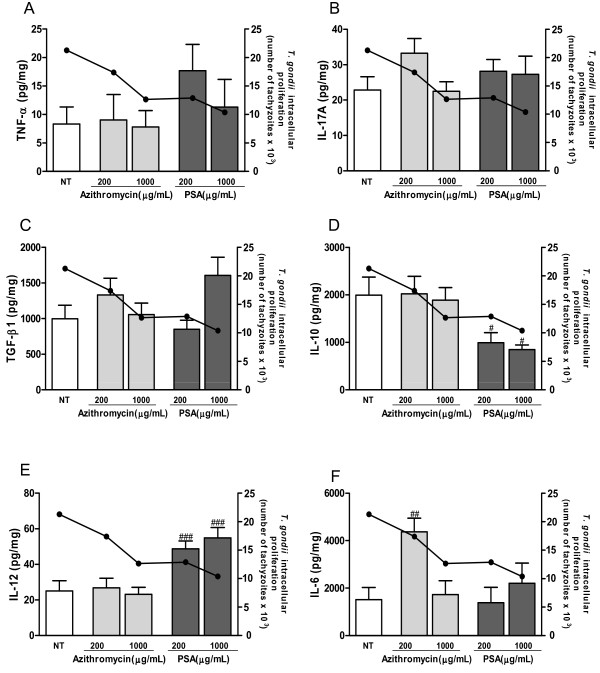
**Cytokine production by *T. gondii *-infected villous explants after treatment with azithromycin or PSA.** Supernatants of villous explants were collected after 24 h of treatment and production of TNF-α **(A)**, IL-17A **(B)**, TGF-β1**(C)**, IL-10 **(D)**, IL-12 **(E)** and IL-6 **(F)** were measured by a CBA or ELISA. The continuous line represents the parasite load. Data are expressed as mean ± SEM. Data were analyzed by ANOVA and Bonferroni multiple comparison post hoc test. ^###^P *<* 0.001; ^##^P *<* 0.01 and ^#^P *<* 0.05 in relation to NT control.

Given the efficiency of both treatments in control of parasitic infection, we next investigated whether antibiotic treatments were able to alter cytokine production by villous explants. Analyses of cytokine levels showed that azithromycin and PSA treatments at 200 and 1000 μg/mL did not alter TNF-α, IL-17A or TGF-β1 production by infected villous explants compared to untreated/infected controls (NT) (Figure 3, A to C; ANOVA, P < 0.001). However, a decrease in the *T. gondii* proliferation in villous explants treated with either drug was observed.

*T. gondii*-infected villous explants produced lower levels of IL-10 and a significant increase of IL-12 after treatment with PSA at both concentrations. Decreased IL-10 levels and increased IL-12 levels matched with reduced parasite load (Figure 3, D and E; ANOVA, P < 0.001).

When infected villous explants were treated with azithromycin at 200 μg/mL*,* an increase in IL-6 levels was observed relative to untreated/infected controls (Figure 3F; ANOVA, P < 0.001). IL-2, IFN-γ and IL-4 levels were not detectable in any culture supernatants.

### *T. gondii* infection increases estradiol, progesterone and HCG + β production in villous explants and antibiotic treatment downmodulates these hormones

**Figure 4:**
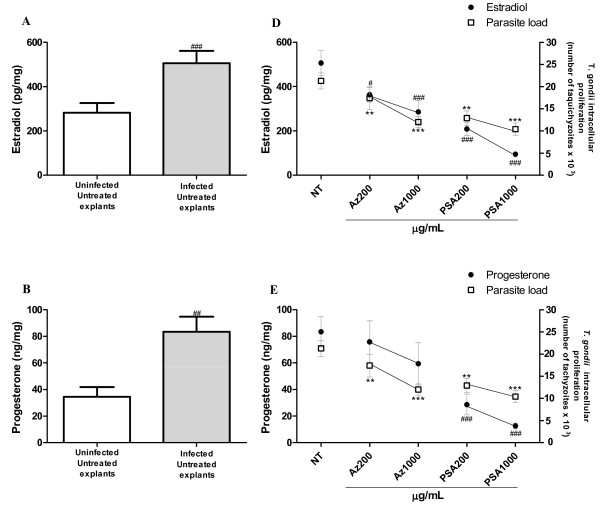
**Detection of sex hormones and parasite load in uninfected and *T. gondii*-infected villous explants, treated or not with azithromycin or PSA.** Estradiol **(A)**, progesterone **(B)** and HCG + β **(C)** were measured by an electrochemiluminescence immunoassay of uninfected or *T. gondii-*infected villous explants. Levels of **(D)** estradiol, **(E)** progesterone, and **(F)** HCG + β in azithromycin- or PSA-treated or untreated villous explants were analyzed in relation to parasite load. Data were analyzed by ANOVA and Bonferroni multiple comparison post hoc test. Comparisons are represented by symbols: parasitism, ^***^P *<* 0.001, ^**^P *<* 0.01, ^*^P *<* 0.05 and sexual hormones, ^###^P *<* 0.001, ^##^P *<* 0.01, ^#^P *<* 0.05 in relation to NT control.

In order to better understand the action of antibiotics on the physiology of villous explants, we conducted a second set of experiments to determine whether *T. gondii* infection is able to alter the hormonal production profile during treatment with azithromycin or PSA. Increased secretion of estradiol, progesterone and HCG + β in supernatants of *T. gondii*-infected villous explants was observed compared to their respective controls (Figure 4, A to C; ANOVA, P < 0.001). However, when infected villous explants were treated with azithromycin, a reduction in the secretion of estradiol and HCG + β was concomitant with a decrease in parasite load (Figure 4, D to F; ANOVA, P < 0.001). PSA treatment of *T. gondii*-infected villous explants induced a significant decrease in estradiol and progesterone compared to infected/untreated controls (Figure 4, D to F, ANOVA, P < 0.001). Simultaneously, there was a reduction in parasite load. In contrast, the effects of PSA on HCG + β secretion showed a significant reduction in the hormone secretion with respect of infected untreated explants, although no difference was observed between the two concentrations tested. However, PSA treatment reduced the parasite load.

### Pretreatment of *T. gondii* tachyzoites with azithromycin or PSA reduces infection in villous explants

**Figure 5:**
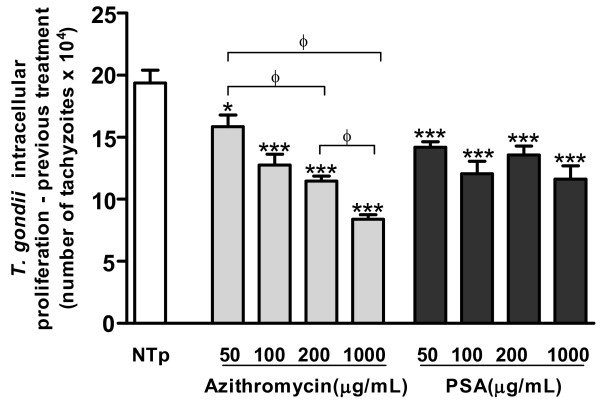
***T. gondii *intracellular proliferation in villous explants infected with pretreated (azithromycin or PSA) parasites.** Proliferation was measured by a colorimetric β-galactosidase assay. The bars represent mean ± SEM. Data were analyzed by ANOVA and Bonferroni multiple comparison post hoc test. ^***^P *<* 0.001, ^**^P *<* 0.01 and ^*^P *<* 0.05 in relation to NTp control. ^Φ^ Comparison between villous explants treated with different concentrations of azithromycin (P < 0.05).

The direct effect of azithromycin or PSA on parasites was subsequently investigated. *T. gondii* 2 F1 tachyzoites were pretreated with different concentrations (50, 100, 200 and 1000 μg/mL) of the two drugs (Figure 5). Azithromycin and PSA pretreatment at all concentrations reduced proliferation of *T. gondii* compared to controls, but a dose-dependent relationship was only observed for azithromycin (Figure 5; ANOVA, P < 0.05), suggesting a direct action on the parasite.

### Production of nitrite

No production of nitrite was detected in supernatants from villous explants in all experiments, suggesting that nitrogen monoxide (NO) is not involved in the reduction of parasite load in this experimental design.

## Discussion

The conventional treatment for toxoplasmosis in pregnant women is based on administration of spiramycin or a combination of pyrimethamine, sulfadiazine and folinic acid (PSA) in cases of fetal infection [23]. Pyrimethamine is a potentially teratogenic antibiotic and should not be used during the first trimester of pregnancy [8]. Thus, it is essential to develop new drugs, as well as new therapeutic approaches, for the treatment of congenital toxoplasmosis. Here, we showed that azithromycin is as effective as PSA treatment of human placental villous infected with *T. gondii*.

Drug dosages used (200 and 1000 μg/mL for both azithromycin and PSA) were selected on the basis of lowest cytotoxicity, which was indirectly assessed by release of LDH. Both azithromycin and PSA were able to reduce *T. gondii* proliferation at both tested concentrations (200 and 1000 μg/mL). These data are in agreement with our previous studies, where azithromycin treatment was able to control *T. gondii* infection in human trophoblast BeWo cells [19]. Additionally, azithromycin also showed a protective role against the ME49 strain of *T. gondii* in rodent *C. callosus*, reducing vertical transmission of the parasite [18]. In the latter study, the number of tachyzoites and bradyzoites of *T. gondii* decreased significantly compared to untreated or PSA-treated *C. callosus*, demonstrating the effectiveness of azithromycin treatment [18]. In another study, azithromycin also reduced *T. gondii* infection in maternal brains and fetal eyes in a *C. callosus* model of congenital toxoplasmosis [27].

Azithromycin can be administered safely during the three trimesters of pregnancy and does not have teratogenic effects [17,28,29]. A recent study demonstrated hopeful results in the treatment of congenital toxoplasmosis by maternal oral administration of azithromycin in addition to sulfadoxine, pyrimethamine and acetylspiramycin in a case of severe symptomatic fetal toxoplasmosis [7].

We observed that *T. gondii* infection reduced significantly the IL-6 production when compared to untreated and uninfected villous explants, while azithromycin treatment in uninfected villous explants did not alter the IL-6 production (data not shown). However, the level of this cytokine was restored when the infected villous explants were treated with azithromycin. In addition, azithromycin treatment did not alter the production of TNF-α, IL-17A, TGF-β1, IL-10 or IL-12 cytokines. Macrolide antibiotics not only have antibacterial activity, but also anti-inflammatory activity and immunomodulatory effects [30]. Previous studies from our group have shown that azithromycin treatment induces an anti-inflammatory response in trophoblastic cells infected by *T. gondii*[19]. However, in the present work, we observed changes only in IL-6 levels, without any variation in the cytokine profile when *T. gondii*-infected villous explants were treated with azithromycin. Some studies have demonstrated the suppressor effect of azithromycin in the IL-6 production. Azithromycin reduced significantly the IL-6 release in macrophages [31-33] and in the serum of pregnant women infected by *Chlamydia trachomatis*[15], evidencing the anti-inflammatory role of this drug in many experimental models. Some undesirable effects of the anti-inflammatory activity of macrolides include human gingival cells when treated with azithromycin or erythromycin showed no inhibition of matrix metalloproteinase 9 or CXCL8 [34]. Furthermore, clarithromycin or azithromycin has not a beneficial effect on CXCL8 release by human whole blood cells and alveolar macrophages [35]. These data suggest that the anti-inflammatory action of azithromycin is dependent on cell type and microenvironment. Furthermore, there is evidence that IL-6 plays an important role in resistance to *T. gondii* infection [36,37]. In addition, this cytokine participates in migration and invasion of trophoblasts into the endometrium [38], inducing release of HCG + β in BeWo cells [39] and other models of human trophoblast [40]. On the other hand, increased levels of IL-6 are involved in several aspects of pathogenesis during pre-eclampsia [41].

Conversely, PSA treatment reduced production of IL-10 and increased IL-12 levels. These results suggest a potential pro-inflammatory effect of PSA treatment, although we did not detect production of IFN-γ. During pregnancy, induction of a type-2 immune response has no effect on the pregnancy; however, induction of a type-1 response by parasitic infection can lead to abortion via alteration of the cytokine profile in the fetal-maternal interface [42]. In murine models of congenital toxoplasmosis, an imbalance in cytokine production and increased inflammatory responses are associated with increased apoptosis and necrosis at the site of implantation, leading to embryonic loss [43].

In the present study, we showed that *T. gondii* infection increased production of estradiol, progesterone and HCG + β in villous explants. Azithromycin did not alter the estradiol, progesterone or HCG + β release in uninfected villous, while PSA increased only estradiol and progesterone production at the same condition (data not shown). Thus, these findings demonstrated that azithromycin did not alter the normal hormone profile. In addition, treatment with azithromycin or PSA was able to reduce secretion of these hormones when compared to infected but untreated tissues. It has been described that for some parasitic diseases, as leishmaniasis, toxoplasmosis and malaria, increased levels of progesterone and glucocorticoid are associated with decreased resistance in murine experimental models [44-46]. In mice, increased morbidity during toxoplasmosis has been associated with estradiol levels, while gonadectomy reduces pathogenesis of toxoplasmosis [3]. A high concentration of progesterone has also been shown to increase susceptibility to *T. gondii* infection during pregnancy by suppressing production of IL-12 and IFN-γ [47] and, therefore, suppressing a type-1 response utilizing the progesterone and glucocorticoid receptors [48]. In the present work, increased production of hormones by *T. gondii*-infected villous explants may be a result of an evasion strategy orchestrated by the parasite. Recent works have demonstrated that hormones are deeply involved in the regulation of parasite load both modulating the host immune-endocrine network and parasite proliferation [49]. Thus we hypothesized an indirect action of the drug treatment inducing lower parasite load that in turn decreased hormones secretion. We showed that *T. gondii*-infected villous explants, treated or untreated, failed to induce NO production. A previous study has demonstrated that human villous explants as well BeWo trophoblast cells infected with *T. gondii* were unable to release NO [21,50]. In our experimental design, reduction of parasite load after antibiotic treatment was likely not related to NO.

Our results also showed that pretreatment of parasites with azithromycin or PSA was able to reduce proliferation of *T. gondii* in villous explants. Azithromycin targets the 70S ribosomal subunit of susceptible microorganisms, such as *P. falciparum* and *P. vivax,* interfering with protein synthesis of the parasites [17]. Additionally, parasites from the Apicomplexa phylum have a plastid called apicoplast, which is a target site for binding of diverse drugs with antiparasitic activity [51,52]. On the other hand, blocking the activities of the plastid does not typically result in immediate death of parasites, including *T. gondii*. In contrast, the parasiticidal activity of plastid inhibitors is only detected when tachyzoites attempt to establish infection within a new host cell [17,53].

## Conclusion

The present study demonstrated that azithromycin is able to control *T. gondii* infection in human villous explants from the third trimester of pregnancy, providing evidence that it may be an effective alternative drug for treatment of congenital toxoplasmosis by reducing the proliferation rate of *T. gondii*. Further clinical studies need to be conducted to validate the present findings and to enable new therapeutic approaches for the treatment of *T. gondii* infection at the fetal-maternal interface.

## Ethical approval

The present study had the approval of the Human Research Ethics Committee of the Universidade Federal de Uberlândia, Brazil. Informed consent from all patients was obtained.

## Abbreviations

PSA: Pyrimethamine, sulfadiazine and folinic acid; RPMI 1640: Roswell Park Memorial Institute medium; FBS: Fetal bovine serum; NT: Infected/untreated villous explants; NTp: Untreated parasites; LDH: Lactate dehydrogenase; CBA: Cytometric bead array; ECLIA: Electrochemiluminescence immunoassay; NO: nitrogen monoxide.

## Competing interests

All authors declare that they have no competing interests.

## Authors’ contributions

All authors contributed in one or more of the following ways: LSCF, BFB and EAVF wrote the manuscript. DAOS and JRM revised the manuscript. LSCF, AOG, and MCS performed the collection of placental tissues and experiments involving infection and treatment. LSCF performed all hormone measurements. LSCF and CMOSA performed all toxicity assays. LSCF and MBA performed all ELISAs and CBA experiments. EAVF, NMS, DAOS and JRM contributed to reagent preparation and performed the quantitative analysis. EAVF, OAMF, NMS, DAOS and JRM provided suggestions and valuable discussion throughout the study. We hereby declare that all authors have read and approved the manuscript.
